# Deep Eutectic Solvents pretreatment of agro-industrial food waste

**DOI:** 10.1186/s13068-018-1034-y

**Published:** 2018-02-12

**Authors:** Alessandra Procentese, Francesca Raganati, Giuseppe Olivieri, Maria Elena Russo, Lars Rehmann, Antonio Marzocchella

**Affiliations:** 1 Istituto di Ricerche sulla Combustione – Consiglio Nazionale delle Ricerche, P.le V. Tecchio 80, 80125 Naples, Italy; 2 Dipartimento di Ingegneria Chimica, dei Materiali e della Produzione Industriale – Università degli Studi di Napoli Federico II, P.le V. Tecchio 80, 80125 Naples, Italy; 3 Department of Chemical and Biochemical Engineering, University of Western Ontario, 1151 Richmond Street, London, ON N6A 3K7 Canada

**Keywords:** Agro-industrial waste, Fermentable sugars, Deep eutectic solvents, Biomass pretreatment

## Abstract

**Background:**

Waste biomass from agro-food industries are a reliable and readily exploitable resource. From the circular economy point of view, direct residues from these industries exploited for production of fuel/chemicals is a winning issue, because it reduces the environmental/cost impact and improves the eco-sustainability of productions.

**Results:**

The present paper reports recent results of deep eutectic solvent (DES) pretreatment on a selected group of the agro-industrial food wastes (AFWs) produced in Europe. In particular, apple residues, potato peels, coffee silverskin, and brewer’s spent grains were pretreated with two DESs, (choline chloride–glycerol and choline chloride–ethylene glycol) for fermentable sugar production. Pretreated biomass was enzymatic digested by commercial enzymes to produce fermentable sugars. Operating conditions of the DES pretreatment were changed in wide intervals. The solid to solvent ratio ranged between 1:8 and 1:32, and the temperature between 60 and 150 °C. The DES reaction time was set at 3 h. Optimal operating conditions were: 3 h pretreatment with choline chloride–glycerol at 1:16 biomass to solvent ratio and 115 °C. Moreover, to assess the expected European amount of fermentable sugars from the investigated AFWs, a market analysis was carried out. The overall sugar production was about 217 kt yr^−1^, whose main fraction was from the hydrolysis of BSGs pretreated with choline chloride–glycerol DES at the optimal conditions.

**Conclusions:**

The reported results boost deep investigation on lignocellulosic biomass using DES. This investigated new class of solvents is easy to prepare, biodegradable and cheaper than ionic liquid. Moreover, they reported good results in terms of sugars’ release at mild operating conditions (time, temperature and pressure).

## Background

A numbers of studies have lately been reported in the literature regarding the conversion of lignocellulosic biomass into biochemicals according to the biorefinery approach [1]. Main steps of the biorefinery approach are: biomass pretreatment [2], hydrolysis [3], fermentation process, bio-products/energy recovery, and concentration. Lignocellulosic feedstock such as dedicated wood cultivation and agriculture residues have several disadvantages: high lignin content, production spread on the territory, competition for arable lands and water sources. The high lignin content requires severe pretreatment conditions (e.g., high temperature and pressure) to effectively remove the lignin. The territory spread biomass production requires high transportation costs for biomass supply chain [4]. On the contrary, waste biomass from agro-food industries are a reliable and readily exploitable resource. The residue streams from agro-food industries are rich in carbohydrates. The industries have to fulfill the food and drink market and manage the residue/wastes of the production process to reduce the environment impact as well as the production cost. From the circular economy point of view, direct residues from these industries exploited for production of fuel/chemicals is a winning issue, because it reduces the environmental/cost impact and improves the eco-sustainability of productions.

Agro-food wastes (AFWs) are characterized by high sugar content and, typically, by low lignin content. AFWs are available almost all year. Their production is spread around the European countries. These production features make AFWs well-suited with the operating and logistic requirements not only for the pretreatment step but also for the entire biorefinery process. Some of the main AFWs produced in Europe are from the following industrial food processes: freezing potatoes, “fresh-cut” fruit, coffee and beer production. As regards the potato industry, about 15 Mt yr^−1^ of potato are processed in Europe according to the European potato processors association [5]. The value of the European fresh-cut fruit and vegetable market is about 3.4 billion euros and fruit accounts for 7% of the market volume in Europe. The total EU fresh-cut fruit and vegetables consumption is about 1.4 Mt yr^−1^ [6]. Residues from “fresh cut” industries are a pressing issue, because about 50% of the raw processed vegetables are discarded and their disposal is particularly expensive [7]. Coffee is the second largest traded commodity after petroleum. The European roasted coffee production is about 2 Mt yr^−1^ [8] and large amounts of by-products are generated in the coffee industry [9]. In particular, coffee silverskin (CS) and spent coffee grounds (SCG) are the main coffee industry residues produced, respectively, during the beans’ roasting and the process to prepare “instant coffee”. Beer is the fifth most consumed beverage in the world after tea, carbonates, milk, and coffee. About 30 Mt yr^−1^ of beer is produced in Europe and the main European beer producers are in Germany, UK, Poland, Netherlands, and Spain at a rate of about 9.5, 4, 4 and 3 Mt yr^−1^, respectively [10].

Several studies focused on pretreatment of agriculture residues such as corncob [11], corn stover [12], and rice straw [13] to produce fermentable sugars. To the author’s knowledge, the use of residues of food and drink processing to produce biochemicals was just addressed to a limited extent [7–14]. In particular, papers regarding sugar recovery by processing coffee silverskin and apple residues are very few [9–15]. The most widely used lignocellulosic pretreatments are energy-intensive processes, because they require high temperature and pressure to remove the lignin [11]. However, recent proposed processes are based on deep eutectic solvents (DESs) that require less energy than the established processes. DESs solubilize the lignin and increase the availability of the cellulose—at low temperature and pressure—for the hydrolysis. DESs are mostly in fluid form composed by two or three ionic compounds able of self-association to form a eutectic mixture [16]. DESs exhibit physicochemical properties close to those of ionic liquids, and lignin solubility included. However, DESs are much more environmental friendly and cheaper than ionic liquids [17]. Potential advantages of DES pretreatments with respect to consolidate processes are reported hereinafter with respect to the corncob exploitation. Zhang et al. [11] reported 54 and 31% of cellulose and lignin content after steam explosion (10 bar, 0.3% H_2_SO_4_). Procentese et al. [18] reported 52 and 10% of cellulose and lignin content after DESs (1 bar, 150 °C, Ch-Cl glycerol) pretreatment. By comparing the energy request for the two processes, Procentese et al. [19] pointed out that the energy required for biomass unit by the DES pretreatment was about twice than that required by the steam explosion. Moreover, the concentration of inhibitors (such as, HMF, acetic acid furfural) is low or even absent after DES pretreatment [18].

Procentese et al. [18] pointed out that glycerol–choline chloride was a potential DES to be used for lignocellulosic materials [18]. Typically, lignocellulosic biomass pretreatment investigations were focused on just one biomass (corncob [18], palm wastes [20], lettuce [19]), on the pretreatment temperature [17, 20], and on processing time [19]. However, investigation also pointed out that a key parameter for the industrial development of the DES pretreatment is the solvent consumption and recovery. Indeed, the reduction of the used DES is an economic prerequisite for the industrial development of the process. Therefore, the biomass to solvent ratio is still an open question for the DES pretreatment processes.

The present work reports the results of a study focused on four AFWs produced from the European food and drink industries: potato peels, apple residues, coffee silverskin (CS), and brewer’s spent grains (BSG). The lignin content of the investigated AFWs ranged between 18% [21, 22] and 33% [23, 24]. The low lignin content of AFWs is typically compatible with the mild conditions for biomass pretreatment adopted by DESs. The investigated AFWS were pretreated with two DESs (choline chloride–glycerol and choline chloride–ethylene glycol) to provide the feedstock for the enzymatic hydrolysis to produce fermentable sugars. Operating conditions of the DES pretreatment were changed in wide intervals. The solid to solvent ratio ranged between 1:8 and 1:32, and the temperature between 60 and 150 °C. The DES reaction time was set at 3 h [19]. The DES-pretreated biomass was hydrolysate according to the NREL protocol. The pretreatment was characterized by two groups of indicators: the first group was assessed after the DES pretreatment, and the second group was assessed after the enzymatic hydrolysis. The first group of indicators included inhibitors, lignin, and sugar content of the recovered pretreated biomass. The second group of indicators included the sugar yield obtained after enzymatic hydrolysis. Operating conditions were tuned to optimize glucose concentration and yield, water consumption, and pretreatment temperature for each AFW. A market analysis to assess the expected European amount of fermentable sugars from the investigated AFWs was also carried out.

## Results and discussion

### Characterization of raw biomass

Selected biomasses were characterized in term of glucan, xylan, arabinan, and lignin content according to the NREL protocol. The starch content was also assessed for all the AFWs. Results are reported in Table 1. Potato peels were characterized by the highest lignin content (33%) followed by coffee silverskin (30%), brewer’s spent grains (22%) and apple residue (19%). As regards the glucan content, the biomasses with the highest value were potato peels and apple residues (31 and 21%, respectively) followed by silverskin and brewer’s spent grains (about 17%). The starch content was quite high for potato peels 23% of starch, quite low for coffee silverskin and BSG (7 and 5%, respectively), and negligible in apple residues.

**Table 1:** Composition of the investigated AFWs

Biomass	Pretreatment			Biomass recovery (%)	Biomass composition (%)^a^				
	DES	Temperature (°C)	Biomass to solvent ratio (g g^−1^)		Glucan	Xylan	Arabinan	AIL^b^	ASL^c^
Potato peels	Raw biomass			–	31.3 ± 0.05	6.0 ± 0.02	1.4 ± 0.01	30.0 ± 0.01	2.9 ± 0.02
	Choline chloride–glycerol	60	1:8	85	31 ± 0.04	6.0 ± 0.02	1.0 ± 0.01	29.9 ± 0.02	2.9 ± 0.01
			1:16	83	35 ± 0.01	5.5 ± 0.01	1.0 ± 0.02	28.5 ± 0.04	2.8 ± 0.02
			1:32	83	37 ± 0.03	5.3 ± 0.02	0.4 ± 0.01	27.5 ± 0.02	2.9 ± 0.02
		115	1:8	80	41 ± 0.03	4.0 ± 0.02	n.d.	26.3 ± 0.01	2.7 ± 0.04
			1:16	75	44 ± 0.05	3.3 ± 0.01	n.d.	24.5 ± 0.03	2.7 ± 0.05
			1:32	75	45 ± 0.05	3.0 ± 0.02	n.d.	23.2 ± 0.01	2.5 ± 0.02
		150	1:8	53	45 ± 0.04	n.d.	n.d.	22.5 ± 0.03	1.9 ± 0.01
			1:16	52	51 ± 0.02	n.d.	n.d.	20.4 ± 0.02	1.9 ± 0.01
			1:32	52	52 ± 0.01	n.d.	n.d.	19.7 ± 0.01	1.8 ± 0.01
	Choline chloride–ethylene glycol	60	1:8	84	31 ± 0.04	6.0 ± 0.04	1.0 ± 0.02	30.0 ± 0.04	2.9 ± 0.02
			1:16	83	35 ± 0.01	5.9 ± 0.01	1.2 ± 0.02	29.5 ± 0.04	2.9 ± 0.01
			1:32	82	36 ± 0.04	5.8 ± 0.02	0.4 ± 0.01	29.0 ± 0.02	2.8 ± 0.02
		115	1:8	80	40 ± 0.05	4.5 ± 0.02	n.d.	27.0 ± 0.05	2.7 ± 0.04
			1:16	76	42 ± 0.04	4.3 ± 0.01	n.d.	25.5 ± 0.03	2.5 ± 0.05
			1:32	74	44 ± 0.01	4.0 ± 0.01	n.d.	24.6 ± 0.02	2.5 ± 0.02
		150	1:8	55	43 ± 0.03	n.d.	n.d.	23.5 ± 0.03	2.0 ± 0.01
			1:16	52	49 ± 0.01	n.d.	n.d.	21.5 ± 0.01	1.9 ± 0.01
			1:32	52	50 ± 0.02	n.d.	n.d.	21.1 ± 0.01	1.8 ± 0.01
Coffee silverskin	Raw biomass			–	17.5 ± 0.05	8.0 ± 0.01	1.6 ± 0.003	27.0 ± 0.02	3 ± 0.01
	Choline chloride–glycerol	60	1:8	80	18 ± 0.01	7.7 ± 0.02	1.2 ± 0.02	26.5 ± 0.02	2.9 ± 0.01
			1:16	80	20 ± 0.01	7.0 ± 0.01	1.0 ± 0.02	25.5 ± 0.01	2.8 ± 0.01
			1:32	80	20 ± 0.02	6.5 ± 0.02	0.6 ± 0.02	25.2 ± 0.02	2.6 ± 0.02
		115	1:8	75	23 ± 0.03	4.0 ± 0.02	n.d.	24.3 ± 0.02	2.6 ± 0.05
			1:16	70	24 ± 0.04	3.3 ± 0.01	n.d.	23.5 ± 0.02	2.2 ± 0.05
			1:32	70	25 ± 0.05	2.0 ± 0.02	n.d.	22.1 ± 0.01	2.0 ± 0.02
		150	1:8	60	30 ± 0.04	1.0 ± 0.01	n.d.	21.5 ± 0.03	1.9 ± 0.01
			1:16	60	32 ± 0.01	1.3 ± 0.02	n.d.	19.4 ± 0.01	1.5 ± 0.02
			1:32	50	33 ± 0.01	0.8 ± 0.04	n.d.	17.7 ± 0.01	1.3 ± 0.02
	Choline chloride–ethylene glycol	60	1:8	80	17 ± 0.04	8.0 ± 0.04	1.0 ± 0.02	27.2 ± 0.04	2.7 ± 0.01
			1:16	79	19 ± 0.01	6.9 ± 0.03	1.2 ± 0.02	26.5 ± 0.04	2.7 ± 0.01
			1:32	79	19 ± 0.03	6.0 ± 0.02	0.8 ± 0.01	26.0 ± 0.02	2.5 ± 0.02
		115	1:8	74	20 ± 0.05	5.4 ± 0.01	n.d.	25.0 ± 0.05	2.7 ± 0.05
			1:16	72	21 ± 0.04	3.3 ± 0.05	n.d.	23.9 ± 0.03	2.5 ± 0.02
			1:32	72	25 ± 0.01	3.0 ± 0.05	n.d.	22.8 ± 0.02	2.5 ± 0.02
		150	1:8	59	25 ± 0.04	1.9 ± 0.01	n.d.	22.1 ± 0.03	2.0 ± 0.01
			1:16	54	27 ± 0.04	1.5 ± 0.01	n.d.	20.0 ± 0.01	1.9 ± 0.04
			1:32	50	27 ± 0.03	0.5 ± 0.04	n.d.	19.2 ± 0.01	1.8 ± 0.04
Brewer’s spent grains	Raw biomass			–	16.8 ± 0.03	16.5 ± 0.02	2.0 ± 0.01	20.0 ± 0.03	1.5 ± 0.02
	Choline chloride–glycerol	60	1:8	81	17 ± 0.04	16.0 ± 0.02	2.0 ± 0.02	20.0 ± 0.02	1.5 ± 0.01
			1:16	80	19 ± 0.01	15.5 ± 0.01	1.0 ± 0.02	19.5 ± 0.01	1.5 ± 0.01
			1:32	80	20 ± 0.03	14.3 ± 0.02	0.5 ± 0.01	17.8 ± 0.02	1.3 ± 0.01
		115	1:8	75	21 ± 0.03	12.3 ± 0.02	n.d.	16.3 ± 0.01	1.3 ± 0.04
			1:16	74	23 ± 0.05	11.0 ± 0.01	n.d.	14.0 ± 0.02	1.1 ± 0.05
			1:32	73	24 ± 0.05	9.9 ± 0.02	n.d.	12.2 ± 0.01	1.1 ± 0.02
		150	1:8	53	25 ± 0.04	8.8 ± 0.04	n.d.	11.5 ± 0.03	0.9 ± 0.01
			1:16	52	27 ± 0.02	8.5 ± 0.01	n.d.	10.4 ± 0.01	0.6 ± 0.01
			1:32	52	27 ± 0.01	8.0 ± 0.01	n.d.	10.0 ± 0.01	0.6 ± 0.01
	Choline chloride–ethylene glycol	60	1:8	80	17 ± 0.04	16.5 ± 0.02	1.9 ± 0.01	20.0 ± 0.04	1.5 ± 0.02
			1:16	80	18 ± 0.01	15.9 ± 0.01	1.2 ± 0.01	19.8 ± 0.04	1.4 ± 0.01
			1:32	79	18 ± 0.04	14.5 ± 0.02	0.4 ± 0.01	19.0 ± 0.02	1.5 ± 0.01
		115	1:8	76	20 ± 0.05	12.9 ± 0.02	n.d.	17.0 ± 0.05	1.3 ± 0.02
			1:16	76	23 ± 0.04	11.8 ± 0.01	n.d.	15.5 ± 0.03	1.1 ± 0.05
			1:32	74	23 ± 0.01	10.5 ± 0.01	n.d.	14.6 ± 0.02	1.1 ± 0.02
		150	1:8	55	23 ± 0.03	9.5 ± 0.02-	n.d.	13.5 ± 0.03	0.9 ± 0.01
			1:16	52	24 ± 0.01	8.9 ± 0.01-	n.d.	12.5 ± 0.01	0.8 ± 0.02
			1:32	52	24 ± 0.02	8.6 ± 0.02-	n.d.	10.8 ± 0.01	0.8 ± 0.01
Apple residues	Raw biomass			–	21.2 ± 0.04	12.2 ± 0.01	2.5 ± 0.04	16.5 ± 0.04	2.0 ± 0.01
	Choline chloride–glycerol	60	1:8	80	22.0 ± 0.04	12.0 ± 0.02	2.0 ± 0.01	15.9 ± 0.02	2.0 ± 0.01
			1:16	81	25.1 ± 0.01	11.5 ± 0.01	2.0 ± 0.02	13.5 ± 0.03	2.0 ± 0.01
			1:32	80	25.9 ± 0.03	11.0 ± 0.02	1.9 ± 0.01	12.9 ± 0.02	1.9 ± 0.02
		115	1:8	75	30.0 ± 0.03	8.0 ± 0.02	0.9 ± 0.01	10.3 ± 0.01	1.7 ± 0.04
			1:16	70	33.2 ± 0.05	7.3 ± 0.01	0.5 ± 0.01	9.5 ± 0.03	1.7 ± 0.01
			1:32	70	34.0 ± 0.05	6.5 ± 0.02	n.d.	8.7 ± 0.01	1.2 ± 0.02
		150	1:8	55	34.1 ± 0.04	6.5 ± 0.01	n.d.	7.5 ± 0.01	1.0 ± 0.02
			1:16	50	35.0 ± 0.02	6.0 ± 0.01	n.d.	6.4 ± 0.02	0.9 ± 0.01
			1:32	50	34.9 ± 0.01	5.9 ± 0.02	n.d.	6.1 ± 0.01	0.8 ± 0.01
	Choline chloride–ethylene glycol	60	1:8	81	21.0 ± 0.04	11.9 ± 0.02	2.0 ± 0.01	16.0 ± 0.01	2.0 ± 0.01
			1:16	82	24.1 ± 0.01	11.0 ± 0.01	1.2 ± 0.02	14.1 ± 0.03	1.9 ± 0.01
			1:32	81	24.2 ± 0.03	11.0 ± 0.02	0.8 ± 0.01	13.7 ± 0.02	1.7 ± 0.02
		115	1:8	76	27.0 ± 0.03	10.0 ± 0.02	0.5 ± 0.01	11.3 ± 0.01	1.4 ± 0.04
			1:16	76	32.2 ± 0.05	8.3 ± 0.01	0.2 ± 0.01	9.5 ± 0.02	1.4 ± 0.01
			1:32	72	32.3 ± 0.05	7.5 ± 0.02	n.d.	8.2 ± 0.01	1.0 ± 0.02
		150	1:8	50	31.1 ± 0.04	7.0 ± 0.01	n.d.	8.1 ± 0.01	1.0 ± 0.02
			1:16	50	33.8 ± 0.02	6.5 ± 0.01	n.d.	7.4 ± 0.01	0.9 ± 0.02
			1:32	50	34.2 ± 0.01	6.5 ± 0.02	n.d.	7.1 ± 0.01	0.9 ± 0.02

Data assessed for raw material and after DES pretreatment are reported

*n.d.* not detected

^a^Percentages calculated on biomass dry weight basis

^b^Acid insoluble lignin

^c^Acid soluble lignin

### DES pretreatment

Table 1 reports the characterization of the investigated AFWs after the DES pretreatment. Pretreated samples of potato peel, coffee silverskin, brewer’s spent grains and apple residues were characterized in term of biomass recovery and composition (glucan, xylan, arabinan, and lignin content).

The biomass recovery, the lignin content, and the pentose-polymers (xylan plus arabinan) content of the pretreated samples decreased with the temperature. In particular, the decreasing of the lignin content ranged between 33% (potato peels) and 62% (apple residues). The increasing of the glucan content was 94% (CS), 67% (potato peels), 66% (apple residues) and 59% (BSG). Although the decrease of the lignin content may be advantageous for the successive processes, the loss of biomass and of pentoses does not suggest operating at high temperature. According to the literature [2], temperature higher than 150 °C was not tested to keep the costs of the whole process low.

The increase of the solvent mass for unit of mass of AFWs is typically favorable to increase lignin dissolution and, as a consequence, sugar content of the pretreated biomass. Indeed, doubling the solvent content from 8 to 16 g per gram of biomass provided an increase in lignin dissolution in the solvent and glucan content in the pretreated biomass, even though the pentose-polymers slightly decreased. However, the further increase of the solvent mass for unit of mass of AFWs (16–32 g_DES_/g_raw biomass_) provided very slight (or negligible) increase of glucan content, because almost no further increase in lignin removal and decrease of pentose-polymers were measured.

The slight advantages from the increase of the solvent mass for unit of mass of AFWs suggest to keep this ratio as low as possible provided that the lignin dissolution in the solvent and glucan content in the pretreated biomass are close to the asymptotic values. Although the aim of the present investigation was to optimize the solvent amount required for the lignin removal, processes to recover and reuse the DES are on the table. As reported in literature [25], DESs can be easily recovered by distillation as pointed out in patents [26]. However, further investigation is required to have a clear scenario of the overall process: from the DES utilization to the DES recovery.

The effects of the temperature and of the solvent mass for unit of mass of AFWs did not changed with the nature of the DESs. The analysis of the results reported in Table 1 points out that the DES made of choline chloride and glycerol provided a slight increase of the delignification process with respect to choline chloride and ethylene glycol.

Results of the DES pretreatment—lignin removal and biomass recovered—may depend on the structure of the investigated biomass. Results could be related to the structure of the investigated biomass. The structure characterization could take advantages from physical and chemical analyses such as FTIR, X-ray, SEM and TEM. Further investigation should be carried out to point out relationships between biomass structure and pretreatment performance.

The comparison of these results with respect to the results reported in the literature is challenging. However, few studies available in the literature are focused on the combination of AFWs and DES pretreatment. Main results regarding the AFWs pretreatment by means of the “classical” processes (e.g., steam explosion, alkaline and extrusion pretreatments) are reported hereinafter. Main results regarding the AFW pretreatment are:

- The extrusion of potato peels at 150 °C produced a glucan content increase of about 2% and a lignin content decrease of about 14% [27];
- CS pretreatment by 0.1 M NaOH produced a glucan content increase of about 22% [28];
- BSG pretreatment by steam explosion at 200 °C and 15.55 bar produced a glucan content increase of about 27% and a lignin content decrease of about 28% [29];
- Apple residues pretreated by steam explosion at 5 bar produced the higher soluble dietary fiber value (29.85%) [30].

Agro-food wastes pretreated by DESs include: corncob [18] and lettuce leaves [19]. Both AFWs were pretreated with choline chloride glycerol at 150 °C for 16 h. The lignin content decrease and the glucan content increase were of about 23 and 67% [18] and 40 and 82% [19], respectively, for corncob and lettuce.

Altogether, reported results point out that sugar recovery and lignin removal produced by means of DES pretreatment is comparable or even higher than that produced by means of the reported pretreatment methods. In addition, the DES pretreatment is characterized by environmentally friendly conditions.

### Inhibitor formation

Potential inhibitors of the enzymatic hydrolysis and of the sugar fermentations produced during the DES pretreatment were analyzed. HMF, furfural, gallic acid, ferulic acid, coumaric acid concentration was measured in the supernatant recovered from the NREL biomass characterization. The supernatant characterization was carried out for the samples produced after the biomass pretreated with DESs under all the operating conditions investigated. HMF and furfural concentration was lower than 1.5 * 10^−2^ g L^−1^. The concentration of gallic, ferulic, and coumaric acid was smaller than the minimum detectable value (1 * 10^−1^ g L^−1^). The measured inhibitor concentrations are in agreement with those previously reported for Ch-Cl glycerol pretreatment applied to corncob [17].

The concentration of the measured inhibitors is lower than the typical threshold of enzymatic hydrolysis and fermentation [25]. Therefore, no detoxification strategy is required after DES-based biomass pretreatment.

### Enzymatic hydrolysis

Table 2 reports the glucose yield referred to the glucane *Y*_e_ (g_glucose_/g_glucan_) and to the pretreated biomass *Y*_1_ (g_glucose_/g_pretreated biomass_) assessed for each pretreated biomass after the enzymatic hydrolysis. The low xylan content measured at high temperature of the pretreatment process (115 and 150 °C) did not address the supplement xylanase to the enzymatic cocktail. As expected, xylose, mannose, and arabinose were not detected in the hydrolysed and were not reported in Table 2. The analysis of the Table 2 points out that both glucose yields increased as the temperature and solid/solvent ratio set during the DES pretreatment (Table 1). As regards the DES couple, the highest glucose yields were measured when choline chloride–glycerol DES was used. In particular, the enzymatic glucose yield (g_glucose_/g_glucan_) measured for biomass pretreated with choline chloride–glycerol was larger than that measured after choline chloride–ethylene glycole pretreatment. The highest values were obtained for apple residues and brewer’s spent grains which were pretreated at 150 °C with biomass to solvent ratio 1:32. The high *Y*_e_ measured for apple residues and brewer’s spent grains could be due to the low lignin content of these residues with respect to potato peel and coffee silverskin.

**Table 2:** Glucose yield after enzymatic hydrolysis of DES-pretreated AFWs

AFW	DES pretreatment			Enzymatic glucose yield, *Y*_e_ (g_glucose_/g_glucan_)	Glucose yield, *Y*_1_ (g_glucose_/g_pretreated biomass_)
	DES	Temperature (°C)	Solid/solvent ratio		
Potato peels	Raw biomass			0.01	0.005
	Choline chloride–glycerol	60	1:8	0.09	0.03
			1:16	0.12	0.04
			1:32	0.16	0.05
		115	1:8	0.25	0.08
			1:16	0.41	0.13
			1:32	0.48	0.15
		150	1:8	0.58	0.18
			1:16	0.74	0.23
			1:32	0.80	0.25
	Choline chloride–ethylene glycol	60	1:8	0.09	0.03
			1:16	0.12	0.04
			1:32	0.12	0.04
		115	1:8	0.22	0.07
			1:16	0.35	0.11
			1:32	0.41	0.13
		150	1:8	0.54	0.17
			1:16	0.67	0.21
			1:32	0.74	0.23
Apple residues	Raw biomass			0.02	0.006
	Choline chloride–glycerol	60	1:8	0.14	0.03
			1:16	0.21	0.04
			1:32	0.23	0.05
		115	1:8	0.47	0.10
			1:16	0.76	0.16
			1:32	0.85	0.18
		150	1:8	0.88	0.18
			1:16	0.92	0.19
			1:32	0.95	0.20
	Choline chloride–ethylene glycol	60	1:8	0.14	0.03
			1:16	0.19	0.04
			1:32	0.19	0.04
		115	1:8	0.38	0.08
			1:16	0.66	0.14
			1:32	0.76	0.16
		150	1:8	0.80	0.17
			1:16	0.85	0.18
			1:32	0.90	0.19
Coffee silverskin	Raw biomass			0.03	0.005
	Choline chloride–glycerol	60	1:8	0.12	0.02
			1:16	0.14	0.02
			1:32	0.16	0.03
		115	1:8	0.23	0.04
			1:16	0.29	0.05
			1:32	0.35	0.06
		150	1:8	0.70	0.12
			1:16	0.83	0.14
			1:32	0.88	0.15
	Choline chloride–ethylene glycol	60	1:8	0.11	0.02
			1:16	0.12	0.02
			1:32	0.13	0.02
		115	1:8	0.17	0.03
			1:16	0.25	0.04
			1:32	0.30	0.05
		150	1:8	0.64	0.10
			1:16	0.76	0.13
			1:32	0.79	0.13
BSG	Raw biomass			0.02	0.004
	Choline chloride–glycerol	60	1:8	0.11	0.02
			1:16	0.16	0.03
			1:32	0.17	0.03
		115	1:8	0.26	0.04
			1:16	0.34	0.06
			1:32	0.38	0.06
		150	1:8	0.76	0.13
			1:16	0.94	0.16
			1:32	0.97	0.16
	Choline chloride–ethylene glycol	60	1:8	0.10	0.02
			1:16	0.14	0.02
			1:32	0.15	0.03
		115	1:8	0.23	0.04
			1:16	0.32	0.05
			1:32	0.35	0.06
		150	1:8	0.64	0.11
			1:16	0.82	0.14
			1:32	0.88	0.15

The analysis of Tables 1 and 2 suggests that severe DES pretreatment—high temperature and large solid to solvent ratio—reduced the amount of recovered biomass but increased the enzymatic digestibility of the carbohydrates. The enzyme accessibility to carbohydrate polymers could be increased for harsh severe operating conditions.

### AFW pretreatment optimization

One of the main pressing issues related to DES pretreatment is the water amount required in the washing step of the pretreated biomass before the enzymatic hydrolysis. Table 3 reports the enzymatic hydrolysis yields *Y*_e_ assessed for tests carried out to assess the effect of the volume of washing water for unit of mass of raw biomass (*V*_w_, mL_water_/g_raw biomass_) on the performance of the process. Data in table refer to all investigated AFWs, pretreated with choline chloride–glycerol at temperature set at 115 and 150 °C. The highest *V*_W_ reported for each set of operating conditions is the minimum volume of water (e.g., minimum number of washing steps) to be used, so that the DES/water phase was completely clear to mark the absence of DES.

**Table 3:** Effect of the extent of washing step on enzymatic hydrolysis of DES-pretreated biomass

AFW	DES	Biomass/solvent ratio (−)	Temperature (°C)	Water consumption *V*_w_ (mL_water_/g_raw biomass_)	Enzymatic glucose yield, *Y*_e_ (g_glucose_/g_glucan_ %)
Potato peels	Choline chloride–glycerol	1:16	115	60	41.9
			*115*	*30*	*35.5*
			150	80	74.2
			*150*	*40*	*67.7*
Apple residues			115	60	76.2
			*115*	*30*	*61.9*
			150	80	92.9
			*150*	*40*	*76.2*
Coffee silverskin			115	60	29.4
			*115*	*30*	*17.6*
			150	80	83.8
			*150*	*40*	*58.8*
Brewer’s spent grains			115	60	34.7
			*115*	*30*	*23.5*
			150	80	94.1
			*150*	*40*	*64.7*

At 115 °C, as the *V*_W_ is halved, the enzymatic glucose yield *Y*_e_ decreases 15–40% with respect to the optimal value: the minimum reduction was measured for potato peels, the maximum reduction was measured for coffee silverskin. The large *Y*_e_ decrease measured for coffee silverskin and brewer’s spent grains may be due to the low glucan content of the raw biomass. Indeed, an extended washing is required to maximize the availability of glucan for the enzymatic hydrolysis. The same behavior may be observed at 150 °C even though the extension of the reduction of *Y*_e_ is less pronounced.

The comparison of the results reported in the present paper with those reported in the literature is not straightforward because—to the authors’ knowledge—no study is reported regarding the optimization of the water consumption during DES pretreatment. The reported results address for a further investigation regarding this issue.

### European fermentable sugars production from AFWs

Table 4 reports the European availability for the investigated biomasses and the expected fermentable sugars production in Europe from these AFWs assessed by processing data of *Y*_2_ yields measured in the present work. Data refers to AFWs pretreated with choline chloride–glycerol at 115 °C and using 30–40 mL_water_/g_raw biomass_ during the washing step (see previous section). The comparison of the data assessed in the present investigation and those reported in the literature is challenging. However, a comparison with the literature is challenging due to the lack of data regarding to the investigated biomass and regarding to the investigated parameters (the water consumption during wash step). To the authors’ knowledge, this is the first investigation carried out paying attention to AFWs pretreatment processes characterized by low energy–water request.

**Table 4:** Expected European fermentable sugar production from investigated AFWs

Wastes	European feedstock availability (Mt yr^−1^)	*Y*_2_ (g_glucose_/g_raw biomass_)	Expected fermentable sugars production (kt yr^−1^)
Potato peel	0.45	0.077	35
Apple residue	0.10	0.091	9
Coffee silverskin	0.2	0.021	3
Brewers’ spent grains	6.0	0.028	170

The largest production of fermentable sugars (170 kt yr^−1^) is expected from BSG after pretreatment with choline chloride–glycerol DES at 1:16 biomass to solvent ratio, 115 °C and rough biomass washing.

## Conclusions

Deep eutectic solvent pretreatment of four different agro-food wastes (AFWs) was successfully carried out. Investigated AFWs were: potato peels, coffee silverskin, brewery’s spent grains, apple residues. Tests were aimed at the selection of optimal operating conditions to maximize sugar production and minimize water consumption. Optimal operating conditions were: 3 h pretreatment with choline chloride–glycerol at 1:16 biomass to solvent ratio and 115 °C.

An analysis of the European agro-food market was carried out to assess the expected fermentable sugar production from the investigated AFWs based on the sugar yields resulting from the experimental investigation. The overall sugar production was about 217 kt yr^−1^ whose main fraction was from the hydrolysis of BSGs pretreated with choline chloride–glycerol DES at the optimal conditions.

## Methods

Chemicals (choline chloride, glycerol, ethylene glycol) and sterile-filtered water were supplied by Sigma Aldrich^®^.

### Raw material, preparation and characterization

Potato peels and apple residues were kindled supplied by a potato processing company and a Spanish fruit juice company, respectively. Coffee silverskin (CS) were kindly supplied by Illy caffè S.p.A. The brewer’s spent grains (BSG) were kindly supplied by an Italian brewery company. The supplied biomass was oven-dried at 40 °C and sieved. Solids collected in the range 1–0.5 mm were stored in sealed plastic bags at room temperature until used.

The raw biomass was characterized in terms of glucan, xylan, arabinan, and lignin content according to the standard protocols of the US National Renewable Energy Laboratory [31].

### DES pretreatment

Two DESs were investigated: (i) choline chloride–glycerol, and (ii) choline chloride–ethylene glycol. The molar ratio of DES components was 1:2 for both couples. The solids were provided by Sigma Aldrich. The DES solutions were prepared by continuously stirring the mixture at 500 rpm in an oil bath at 80 °C until homogenous colorless liquids formed. The raw biomass was mixed with the DES under pre-set operating conditions.

The investigated operating conditions were: (reaction time) 3 h according to the results reported by Procentese et al. [18]; (temperature) 60, 115, and 150 °C [32]; (solid to solvent ratio) 1:8, 1:16, and 1:32 [18].

### Pretreated biomass recovery

The pretreated biomass was recovered by centrifugation. The biomass/DES suspension was mixed with sterile-filtered water to wash the biomass: the two phase suspension (the biomass and the DES/water phase) was centrifuged (3 min at 5000 rpm) to recover the biomass. The washing step was repeated until the DES/water phase was completely clear to mark the absence of DES. The wet slurry was dried at 38 °C until constant weight was reached.

The percentage biomass recovery (*R*) was calculated as the ratio between the dry weight of pretreated biomass (*B*_PT_) and the dry weight of raw material (*B*_RAW_):

1 $$ R = \, B_{\text{PT}} /B_{\text{RAW}} $$

The pretreated biomass was characterized in terms of glucan, xylan, arabinan, and lignin content according to the standard (NREL) protocols of the US National Renewable Energy Laboratory [26].

### Analytical methods

Glucan, xylan, and lignin content of biomass samples (raw and pretreated) were determined by quantitative saccharification upon acid hydrolysis and subsequent HPLC and gravimetric analysis, based on standard NREL protocols [31]. The concentration of glucose and xylose was quantified by high-performance liquid chromatography (Agilent 1260 Infinity HPLC) using an 8 µm Hi-Plex H, 30 cm 7.7 mm column at room temperature and a refractive index detector. Deionized water was used as mobile phase at a flow rate of 0.6 mL min^−1^.

Analysis of each biomass sample was carried out in triplicate.

The measurement of the concentration of potential enzymatic and fermentation inhibitors was also carried out: hydroxymethyl-furfural (HMF), furfural, gallic acid, ferulic acid, coumaric acid were quantified by high-performance liquid chromatography (Agilent 1100 system Palo Alto, CA). Inhibitors were separated by means of Luna C18 column (5 µm 250 × 4.6 mm) at room temperature and optically detected at 276 nm. Mixtures of formic acid 0.1% vol and pure methanol were used as mobile phase with the following solvents profile: 20 min ramp from 0 to 1.2 mL min^−1^ flow rate and from 5 to 30% of methanol, 40 min ramp from 1.2 to 1.5 mL min^−1^ flow rate at constant value 30% of methanol.

### Enzymatic hydrolysis

The enzymatic hydrolysis was carried out according to the procedure proposed by Procentese et al. [19]. The commercial enzyme cocktail Cellic CTec2 (kindly supplied by Novozyme) and Amylases from Megazyme were used. The cellulase activity was adjusted to 142 FPU mL^−1^. The hydrolysis was carried out in 0.1 M sodium citrate buffer (pH 4.8) supplemented with 80 µL tetracycline and 60 µL cycloheximide to prevent microbial contamination. 100 mL glass bottles were incubated at 50 °C and kept under agitation on a rotary shaker (Minitron Incubator Shaker-Infors HT) at 180 rpm for 60 h. The CTec2 loading was set at 15 mg_enzyme_/g_glucan_ according to the glucan content assessed on the raw biomass, the amylases loading was fixed at 10 U/g_pretreatred biomass_. The solid loading was set at 10% (w/v). The enzymatic solution was sampled, centrifuged, filtered, and analyzed to assess sugar concentration at fixed time intervals.

Hydrolysis tests were carried out in duplicate.

As reported, the enzymatic hydrolysis requires the washing of the DES-pretreated biomass and the water consumption is a pressing issue of the process. A campaign of enzymatic hydrolysis tests was carried out by tuning the water used to wash the DES-pretreated biomass. In particular, the volume of washing water for mass unit of raw biomass (*V*_w_, mL_water_/g_raw biomass_) assessed for the DES pretreatment optimal conditions was cut by half. Results of the enzymatic digestibility of pretreated biomass were compared with those assessed for the tests carried out with extended biomass washing.

Enzymatic glucose yield, *Y*_e_, (g_glucose_/g_glucan_) was calculated as the ratio of the glucose produced during the enzymatic hydrolysis and the glucan content in the raw biomass.

The glucose yield referred to the pretreated biomass, *Y*_1_, (g_glucose_/g_pretreated biomass_) was calculated as the ratio between the glucose produced by the enzymatic hydrolysis and the pretreated biomass processed during the enzymatic hydrolysis. The glucose yield referred to the raw biomass, *Y*_2_, (g_glucose_/g_raw biomass_) was calculated as:

2 $$ Y_{2} = Y_{1} * \, R $$

### Assessment of the European fermentable sugars production

#### Potato peels

The main by-products of the potato processing are: potato peel (around 3%), fresh rejected potato (3–4%), starch (3%), and fried rejected potato (2–3%). These residues are characterized by a high carbohydrate content: a source of fermentable sugars. The European potato peels availability was calculated as the product of the potato mass processed every year and the potato peel fraction (3%).

#### Apple residues

According to the reported data, the European apple residue availability was calculated as the product between the total EU fresh-cut fruit (equal to the residue production rate) and vegetables consumed every year and the fruit contribution (approximated as 7% of the mass market volume).

#### Coffee silverskin

Coffee silverskin is about 4.2% of coffee beans and the valorisation of this waste according to the biorefinery concept could be a contribution for many industries for the development of circular economy. Carbohydrates content of CS ranges between 34.6 and 80.5% [21]. Therefore, CS could be used for fermentable sugars production. The year European CS availability was calculated as the product between the total EU processed coffee beans every year and the CS fraction (4.2%).

#### Brewer’s spent grains

Brewers’ spent grains (BSGs) are the residues of the beer production and it is about 20% of the beer produced. BSGs are characterized by high sugar concentration and may be used as feedstock to produce fermentable sugars. The European BSG availability was calculated as the product between the total EU beer production per year and the BSGs fraction (20%).

The fermentable sugar production for each waste (*S*_*i*_) was calculated as following:

3 $$ S_{i} = \omega_{i} \times Y_{1} $$

where *ω*_*i*_ is the European availability (Mt yr^−1^) for each waste *i*, and *Y*_1_ (g_glucose_/g_raw biomass_) is the yield of glucose per gram of raw biomass.
